# Unlocking the potential of cultivated meat through cell line engineering

**DOI:** 10.1016/j.isci.2024.110877

**Published:** 2024-09-06

**Authors:** Camilo Riquelme-Guzmán, Andrew J. Stout, David L. Kaplan, Joshua E. Flack

**Affiliations:** 1 Biomedical Engineering Department, Tufts University Center for Cellular Agriculture, Tufts University, Medford, MA, USA; 2 Deco Labs, Inc., Boston, MA, USA; 3 Department of Biotechnology, Delft University of Technology, Delft, the Netherlands

**Keywords:** Applied sciences, Biotechnology, Food biotechnology

## Abstract

Cultivated meat has the potential to revolutionize food production, but its progress is hindered by fundamental shortcomings of mammalian cells with respect to industrial-scale bioprocesses. Here, we discuss the essential role of cell line engineering in overcoming these limitations, highlighting the balance between the benefits of enhanced cellular traits and the associated regulatory and consumer acceptance challenges. We believe that careful selection of cell engineering strategies, including both genetic and non-genetic modifications, can address this trade-off and is essential to advancing the field.

## Graphical abstract

**Figure:**
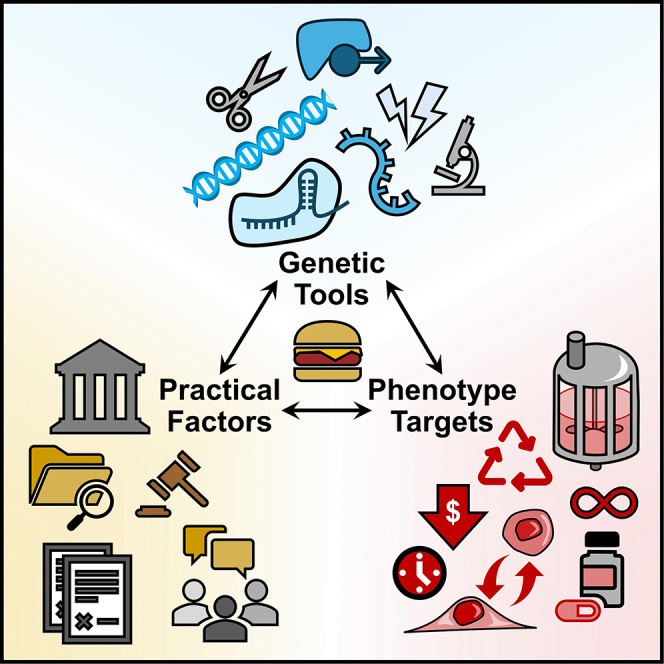

## Introduction

Cultivated meat (also referred to as “cultured” or “cell-based” meat) has emerged as a promising innovation to address modern challenges in food production, distribution, and security. These technologies aim to use large-scale cell culture and bioengineering principles to decouple meat production from the rearing and slaughtering of livestock, thereby mitigating the negative environmental, ethical, and health externalities of animal agriculture.^1^ Manufacturing cultivated meat involves isolating animal cells, controlling their proliferation, differentiation, and maturation into relevant tissues (primarily muscle and fat), and assembling these into a final food product. From a cell biological perspective, this requires accurate, efficient, and cost-effective manipulation of stem cells through complex biological processes *ex vivo*, necessitating the precise fine-tuning of cellular functions at each step of the bioprocess.^2^ Using starting cells that are optimally suited to this manipulation is thus of the utmost importance if cultivated meat technologies are to compete with their traditional counterparts in terms of scale, price, and taste. Across the field, various cell types have now been investigated for their robust proliferative capacity (e.g., embryonic and induced pluripotent stem cells), or for their ability to readily differentiate into relevant tissues (e.g., skeletal muscle-derived satellite cells and fibroadipogenic progenitors).^3^ However, none of these cell types or cell lines currently possess the full array of phenotypes necessary for cost-effective, industrial production.

## Animal cells are poorly suited to industrial bioprocesses

Immortalized cell lines are generally considered a requirement for the generation of huge amounts of edible tissue from a stable, robust bioprocess.^4^ Immortalization of animal cells is well studied, and various methods have now been employed for cultivated meat cell line engineering, including targeted overexpression of key factors (such as CDK4 and telomerase)^5^ and the “spontaneous immortalization” of cells that arises naturally (though unpredictably) in some long-term cultures.^6^^,^^7^ However, while this trait is certainly necessary for a robust bioprocess, and thus an appropriate focus of research, it is alone insufficient.

**Figure 1:**
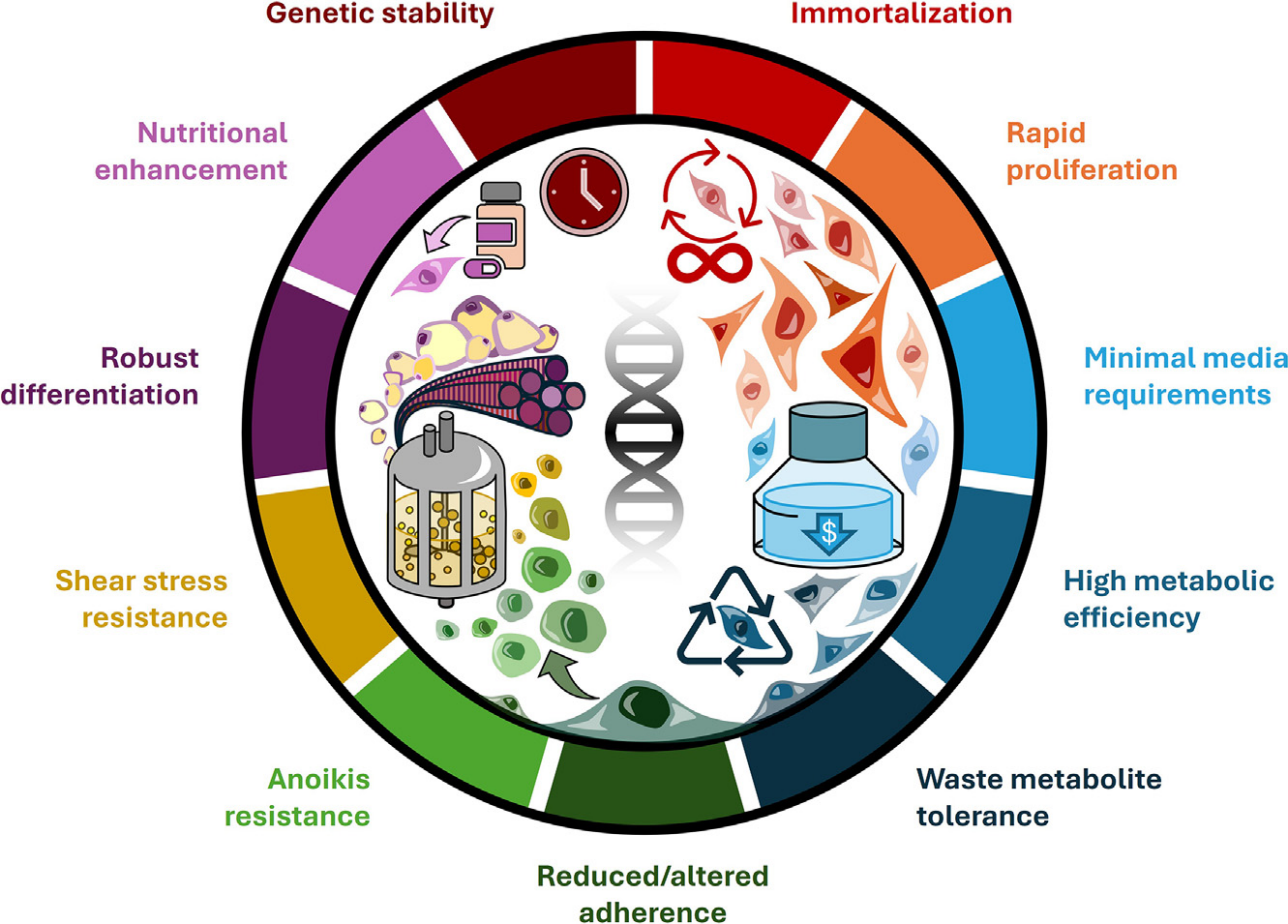
Array of desirable cellular phenotypes for cultivated meat production Cell line engineering can be used to confer a diverse array of phenotypes in mammalian cells relevant for industrial-scale cultivated meat bioprocesses. These phenotypes can not only enhance cellular traits, reducing production complexity and cost, but also provide new functions to cells, creating products with additional value.

Numerous additional phenotypes will be necessary to enable production at the cost and scale required to compete with commodity products (Figure 1, Table 1). Examples include rapid proliferation in low-cost culture medium, viability in high-density suspension culture, metabolic efficiency, waste metabolite tolerance, and shear stress resistance. Such cellular traits can help circumvent major bottlenecks in current cultivated meat bioprocess design, including cell density limitations, medium cost, and requirements for complicating bioprocessing aids, such as microcarriers.^8^ In addition, cultivated meat cell lines should be genetically stable, in order to ensure consistency in downstream processes and final products and retain differentiation capacity even after a lengthy proliferation phase (Figure 1). Although many of these traits are observed in bacterial and yeast strains developed for industrial bioprocesses,^9^^,^^10^ they are rarely found in animal cells (where doubling times are an order of magnitude higher), and the increased cost and complexity of any bioprocess that needs to accommodate cell lines lacking these phenotypes will severely limit the utility of the technology.^8^ However, in many cases, the genetic underpinning of such phenotypes remains unclear, even for mammalian cells. For other species of interest, including fish and crustaceans, our understanding is even more limited.^11^

**Table 1:** Desirable phenotypes for cultivated meat cell lines, with potential genetic engineering targets and strategies that could yield such outcomes

Desired phenotype	Gain of function (overexpression/constitutive activation)		Loss of function (knockout or knockdown)	
	Targets with data in cultivated meat applications	Possible targets based on known biological effects	Targets with data in cultivated meat applications	Possible targets based on known biological effects
Immortalization	• Telomere maintenance (TERT)^5^ • Cell cycle regulators (e.g., CDK4)^5^ • Yamanaka factors (Oct3/4, Sox2, Klf4, c-Myc)^29^	• Immortalizing cisgenes (e.g., c-Myc, Cyclin D1) • Transforming transgenes (e.g., SV40 T-antigen, HPV E6/E7)	• CDK inhibitors (p16, p18, p21, p53)^30^	• Major tumor suppressor genes (e.g., Rb1, PTEN)
Reduced cost/complexity of culture medium	• Growth factors (e.g., (FGF2,^19^ IGF-1^31^) • Glucose transporters (GLUT1–4)^32^ • Key signal transducers (e.g., constitutively active Ras)^19^ • Receptors (e.g., constitutively active Insulin receptor)^32^	• Growth factors/receptors (e.g., HGF, HGFR) • Media components (e.g., transferrin) • Downstream signaling (e.g., Akt, ERK, constitutively active Ras, Raf)		• Downstream signaling inhibitors (e.g., Spry)
Metabolic efficiency and waste tolerance	• Glutamine synthetase (GS)^33^	• Lactate/ammonium reduction (e.g., PYC2, GLUT5, PC)		• Lactate production (e.g., LDHA)
Suspension culture	• Integrin-linked kinase (ILK) • Pyruvate dehydrogenase kinase (PDK1) • Transcription factors (e.g., TBX2, Pax3) • Signaling kinases (e.g., B-Raf, Src) (all^32^)	• Cholesterol metabolism (e.g., MSMO1, HMGCS1, IDI1, NPCL1, INSIG1) • Cell-cell and cell-matrix interactions to promote aggregate formation (e.g., cadherins)	• Cell cycle regulators (e.g., PTEN) • Caspases (e.g., CASP3, CASP8) • Cell adhesion factors (e.g., CDH1, ITGB1) (all^30^)	• Suspension adaptation genes from HEK293 (e.g., RARG, ID1, ZIC1, LOX, DHRS3) • Cell-cell and cell-matrix receptors to reduce surface adherence (e.g., integrins) or reduce aggregate formation (e.g., cadherins) if desired.
Improved viability in high density cell culture	• Yes-associated protein (YAP1)^31^	• Anti-apoptotic genes (e.g., BCL2, BCL-xL)		• Pro-apoptotic genes (e.g., BAX, BAK, caspases)
Robust differentiation	• Myogenic transcription factors (e.g., MyoD,^34^^,^^35^ myogenin^36^) • Adipogenic transcription factors (e.g., PPARγ)^37^	• Myogenic factors (e.g., Myf5) • Adipogenic factors (e.g., CEBPα/β)		• Myogenic inhibitors (e.g., myostatin) • Adipogenic inhibitors (e.g., SMAD3, FOXO1)
Nutrition and flavor enhancement	• Antioxidant synthesis (phytoene synthase [CrtB], phytoene desaturase [CrtI], lycopene cyclase [CrtY])^20^ • Heme-containing proteins (e.g., myoglobin)^38^	• Fatty acid desaturases (e.g., Fat1, Fat2) • Phytonutrient synthesis (e.g., DFR, ANS, UFGT) • Skeletal muscle proteins (e.g., myosin heavy chain)		• Deleterious nutrient synthesis genes (e.g., BBOX) • Off-flavor precursor synthesis genes (e.g., FADS1, ALOX5) • Allergen synthesis (e.g., GCTA1)

Genuine upscaled cultivated meat will thus only become reality with the development of highly optimized cell lines, possessing an array of enhanced phenotypes which can be cultured and differentiated efficiently at large scales, while also meeting the price, taste, nutrition, and sustainability criteria necessary to achieve meaningful market penetration. In this regard, animal cell engineering in the biopharmaceutical industry, including the optimization of Chinese hamster ovary (CHO) cells for protein production and development of next-generation chimeric antigen receptor (CAR)-based cell therapies, can be a powerful source of inspiration for both phenotypes of interest, and the tools to engineer them.^12^^,^^13^^,^^14^^,^^15^ For example, recent studies have improved mammalian cell growth dynamics and subdued apoptotic pathways for optimized biomanufacturing of CHO cells,^16^ or modified gene expression for reduced CAR-T cell exhaustion.^17^ Even so, both the scale and economic requirements for cultivated meat remain unprecedented in terms of animal cell culture.^1^^,^^18^ We therefore suggest that, no matter the starting cell type, harnessing *cell-autonomous* mechanisms through cell engineering strategies is essential for the development of optimal cell lines for cultivated meat production.

## Genetic engineering is a powerful tool for improving cell lines

**Figure 2:**
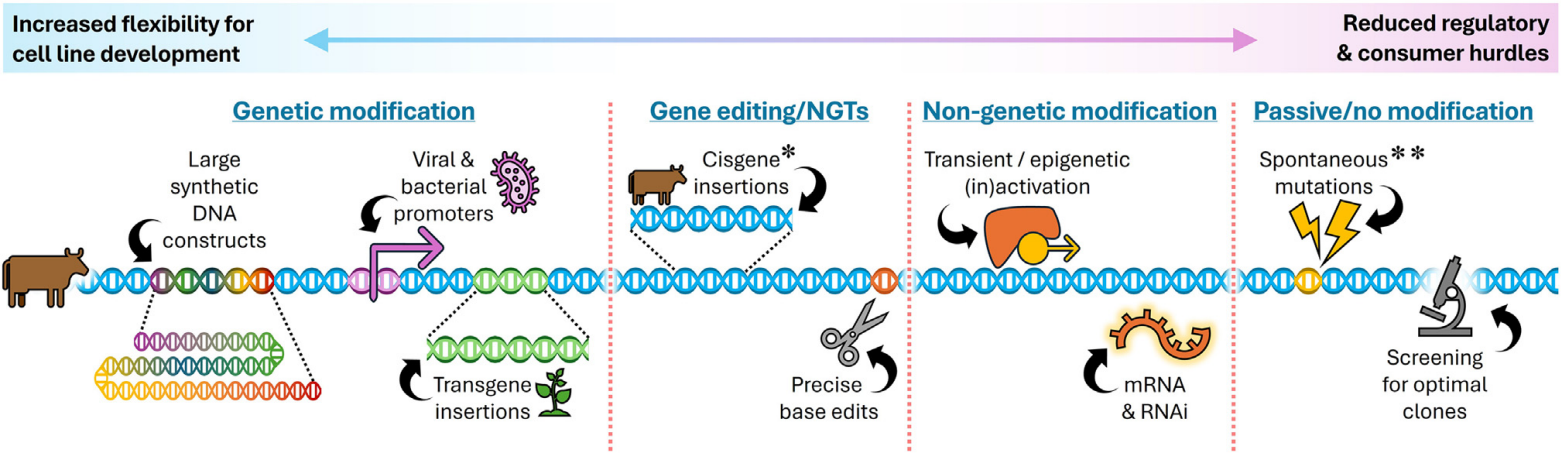
Genetic engineering tools and their application to cultivated meat Trade-off between the phenotypic flexibility afforded by more drastic genetic modifications with higher percieved consumer and regulatory risk (left) and less flexibility but reduced consumer and regulatory risk (right). ∗indicates currently approved in a cultivated meat product in the US (cisgenic TERT immortalization), ∗∗indicates currently approved in a product in US and Singapore (spontaneous immortalization). NGTs, new genomic techniques.

Recent years have seen rapid evolution in the development of genetic engineering technologies and increasing availability of highly sophisticated gene editing tools, driven largely by the cell and gene therapy industries. However, many of these tools can equally be applied to cultivated meat (Figure 2). Several proof-of-concept studies have now been published in the field, including the generation of immortalized cell lines,^5^ removal of growth factor requirements from serum-free proliferation medium formulations,^19^ and the enhancement of nutritional value.^20^ A number of patents from the industry, targeting various cellular traits including reprogramming, proliferation, and differentiation, have also now been filed.^3^^,^^21^^,^^22^

Despite this backdrop, little attention has focused on the use of genetic engineering for cultivated meat development, and the generation of new phenotypes through slower “spontaneous adaptation” of cells is generally preferred by industry.^7^^,^^23^ Relying on random genetic mutation (either spontaneous, or induced by DNA-damaging agents) for the acquisition of phenotypes, however, poses substantial limitations. Specifically, it is extremely inefficient (especially for cells from larger animals), requires acceptance of a large number of unknown genetic changes, and creates long-term reproducibility issues between cell lines.^6^^,^^24^ Moreover, many phenotypes cannot realistically be expected to arise spontaneously at all, either because they require numerous specific mutations or because they do not give rise to a proliferative advantage allowing them to overgrow other cells. The possibility of combining multiple phenotypes acquired in this way also becomes vanishingly small as the low probabilities of individual changes compound. Genetic engineering strategies thus bring an opportunity to enhance, reduce, or optimize cellular processes in a straightforward, reproducible, and combinatorial fashion, enabling the acquisition of much-needed phenotypes for robust mass production of cultivated meat (Figure 2).

Historically, genetically modified organisms (GMOs) for food applications have been produced via integrative techniques. Fast, flexible, and powerful methods including viral-, transposon-, and integrase-mediated gene transfer are commonly used to insert large sequences, including open reading frames, regulatory elements, or even combinations of multiple genes. These techniques can be either “transgenic” (involving transfer of genetic material from a different species) or “cisgenic” (involving transfer of material already found within the genome of interest). In a cultivated meat context, these tools have enabled, among others, the development of transgenic bovine muscle satellite cells that produce carotenoids (important antioxidants found in many vegetables),^20^ and cells capable of inducible differentiation (which can be cisgenic or transgenic, depending on the origin of the genes and promoters used).^25^ Overexpression of master transcription factors is likely to drive faster and more efficient differentiation than that achievable through tuning of medium formulations, especially for pluripotent cell types.^26^ Genetic engineering tools can also drive the acquisition of many other phenotypes (see Figure 1 and Table 1), such as suspension growth.^27^ However, whether cis- or transgenic approaches are used can have important regulatory implications, and it is thus key that researchers carefully consider the origin of genes and genetic systems they plan to use. Of note, the US Food and Drug Administration has already approved a cultivated chicken product produced from cisgenically immortalized cells for human consumption, establishing a precedent for use of these technologies.^28^

While these classical strategies generally rely on the stable introduction of foreign genetic material, more recent gene editing technologies (including clustered regularly interspaced short palindromic repeats [CRISPR]-Cas, transcription activator-like effector nucleases [TALENs], and zinc-finger nucleases [ZFNs]) can now be used to create stable changes to the *native* genome, further expanding the cell line engineering toolbox. These tools can be programmed to generate loss-of-function knockouts through DNA double-strand breaks at specific locations, thereby deleting expression of specific genes and allowing cells to bypass genetic circuits with undesirable functions. For example, knockout of p15 and p16 (two cell cycle regulatory proteins) increased the proliferation of muscle progenitors,^21^ whereas Igfbp4 and Aqp1 knockouts favored the adaptation of CHO cells to suspension culture,^39^ both highly sought after phenotypes for cultivated meat production. Other mechanisms for promoting suspension growth, including evasion of anoikis, might also be possible in a cultivated meat context, for example via the deletion of caspase genes.^30^ Targeted gene knockouts could also prove useful for the development of other phenotypes, such as waste metabolite tolerance or improved stress resistance (see Table 1). Importantly, CRISPR-Cas complexes can be supplied transiently (for example as mRNA or mRNPs), minimizing the risk of genomic insertions or off-target effects. Furthermore, the RNA-guided nature of CRISPR methodologies means they can be employed in forward screens for phenotypes where the underlying genetics may be unclear.^40^ Most critically, the lack of foreign DNA means that these *gene editing* strategies are often viewed differently from traditional genetic modification (GM) by regulatory authorities (see next section).^41^

Next-generation gene editing tools, such as CRISPR base and prime editors, have recently emerged from the gene therapy space.^42^ These tools build upon the machinery of the first-generation gene editing systems, but have now opened the door to extremely precise genome rewriting in the absence of double-strand breaks (greatly reducing off-target effects^43^). Gene knockouts can be easily created, for example through nucleotide substitutions that introduce premature stop codons or disrupt splice sites.^44^^,^^45^ Moreover, site-directed mutagenesis through single or multiple amino acid substitutions opens the door for the development of proteins with altered or enhanced gain-of-function mutations, while minimizing the size of the genetic alteration needed (Table 2). Editing of cell surface receptors, signal transducers, or transcription factors, for example, might result in enhanced phenotypes in terms of reduced medium requirements, increased signaling responses, or accelerated differentiation, although responses are likely to be highly context dependent (Table 1). Similar strategies could also be used for transdifferentiation, so that robustly growing cell lines (such as fibroblasts) can be efficiently converted into mature muscle cells following expansion.^46^^,^^47^ Precise editing of promoter sequences of key regulatory proteins could also be a viable strategy to drastically change cellular behavior with minimal genetic change.^48^ Along related lines, recently developed epigenetic editing tools (such as “CRISPRi”) fuse chromatin-modifying enzymes to inactivated Cas9, resulting in stable gene silencing that is heritable across numerous cell divisions, hence driving changes in transcriptional activity *without* permanent modifications to DNA sequence.^49^ Such techniques may allow flexible cell line engineering while avoiding regulatory hurdles associated with more traditional genetic engineering or gene editing techniques.

**Table 2:** Applicable tools for cultivated meat cell line engineering

Effect	Insertions (including cisgenic)				Targeted edits	Non-genome modifying		Random mutagenesis
Category	Transposons	Viruses	Recombinases	Homology- directed repair (HDR)	Indels and controlled edits	Epigenetic control	RNA-based control	Chemical or physical mutagens
**Tool or Technique** (prior or anticipated expiration of an early patent)	Sleeping Beauty (2018)^50^	Retrovirus (2001)^51^	PhiC31 (2019)^52^	CRISPR-Cas (2033)^53^	Nuclease editing (see HDR)	Transcriptional regulation (2016)^54^	mRNA (2008)^55^	Ionizing radiation (N/A)^56^
	PiggyBac (2017)^57^		Cre (2007)^58^	ZFNs (2026)^59^	Prime editing (2040)^60^	DNA (de)methylation (2019)^61^	siRNA (2018)^62^	Nickel chloride (N/A)^56^
	Tol2 (2021)^63^	Lentivirus (2014)^64^	FLP (2014)^65^	TALENs (2030)^66^	Base editing (2034)^67^	Histone (de)acetylation (2019)^61^	miRNA (2023)^68^	Fast neutron bombardment (N/A)^56^
	Minos (2012)^69^		Hybrid recombinases (2027)^70^	Bridge RNA (TBD)^71^	Click editing (TBD)^72^	Chromatin looping (2033)^73^	Cas13 (Pending)^74^	*N-*methyl-*N-*nitrosourea (N/A)^56^
***Notes***	*Less controlled insertion loci*	========>		*More controlled insertion loci*	*Achievable with CRISPR*, *TALENs*, *ZFNs*	*Effects can be semi-stable for generations*	*Transient effects*	*Random mutations*

Expiration date of an exemplary patent covering usage in animal cell line engineering is provided for each tool or technique as a means of framing the current IP landscape (though this does not represent a complete picture, and other patent protections could exist). “TBD” indicates no patents have yet been published; “Pending” indicates that a pending application exists; “N/A” indicates no patents have been found, and that published examples are older than the standard patent lifespan.

## Regulatory, commercial, and intellectual property challenges

Despite the fact that many of the phenotypes discussed are considered important by a significant portion of cultivated meat companies, there is still reluctance in the field to adopt genetic engineering techniques, largely because of regulatory complexity and perceived consumer acceptance concerns.^23^ However, this severely limits the possibility for the phenotype acquisition that is required for these technologies to become viable in the long term. We suggest that rather than adopt a binary view of genetic engineering (i.e., GMO or non-GMO), the reality is a broad spectrum of techniques from which companies in different geographies and with different bioprocess philosophies can select the optimal trade-off between flexibility for cell line engineering and consumer/regulatory acceptance in their case (Figure 2). In regions open to broad application of genetic engineering for food (such as the US), synthetic biology techniques could promote the creation of complex pipelines that accelerates advancement of the field. In some of the more GM-skeptical geographies, cisgenic gene editing is not considered genetic modification (a subtlety that is often overlooked), and these techniques may thus offer a “sweet spot” between phenotypic flexibility and regulatory concerns. Even in the most strict regulatory regimes, manipulation of cells with mRNA or protein expression is usually possible without the final products being considered genetically modified, although this is likely to pose additional challenges, since it would require biological effects that persist long after transient expression of the reprogramming factors in order to be economically viable.

Importantly, these regulations are themselves evolving. The development of precision gene editing technologies means that gene-edited cells are now often indistinguishable from those derived by traditional methods (such as random mutagenesis and selective breeding), encouraging calls for updates to the legal frameworks established for genetic modification in the food industry, particularly for the development of new crop variants.^75^ In the EU and New Zealand, for example, discussions are currently ongoing regarding the current strict definitions of gene-edited foods as genetically modified.^76^ It should be noted, however, that many of the proposed new regulations are focused on crop engineering, and specific legislation for products produced from animal cells might be necessary in the near future. While this may encourage stakeholders in the cultivated meat space to embrace gene editing, it is nevertheless important to bear in mind that introducing multiple modifications to cells, or combining different methods, might still complicate approval processes. For example, creating more complex genetic circuits, such as inducible promoters, would be extremely powerful for cultivated meat processes (e.g., for induction of differentiation^77^^,^^78^), but is likely to be challenging within the boundaries of cisgenic gene editing. However, concepts based on heat shock or hypoxia transcriptional responses might be promising in this respect, given that they are easy to induce within a bioreactor-based bioprocess and elicit strong induction of gene expression.^79^^,^^80^ Inducible systems are important tools for therapeutic applications, and a similar outcome for cultivated meat is feasible if accompanied by appropriate regulation.^81^

In addition, both private and academic institutes will need to consider how the intellectual property landscape can affect freedom to operate for a given cell line engineering strategy. For instance, while CRISPR-Cas-based technologies are undeniably powerful and flexible, their relative novelty brings restrictions in the form of a large number of active patents, which will not expire for over a decade, and which pose potentially complex routes for licensing.^82^ This will incur additional costs that could hinder the application of CRISPR-based strategies for cultivated meat, or even limit their use entirely. As an alternative, researchers could look toward older technologies that are moving (or have already moved) into the open domain, such as the PhiC31 integrase^83^ or Mad7 nuclease,^84^ or consider new open-access tools that are being developed.^85^

## Outlook

Advancements in cell line development must be accompanied by appropriate regulations to support food safety, protect consumers, and establish clear boundaries for the industry. However, most cell engineering work to date has been conducted by private companies, making it difficult to accurately assess the state of the industry and increasing the risk of redundant efforts, especially concerning cell immortalization.^86^ Nevertheless, the door remains open for creation of an innovative space for collaborative, open-access research with a far greater impact in the field. Indeed, when thinking about more complex phenotypes and sophisticated engineering strategies, which are not yet ready for immediate application in industry, academic institutions would seem to be ideal settings for responsible research and innovation.

Mammalian cells in their unmodified state are poorly suited to industrial bioprocesses, especially those trying to operate at extremely low cost. However, genetic engineering tools and methods are now improving to the place where these limitations can be tackled in earnest. The *in vitro* nature of cultivated meat production provides a uniquely fertile and exciting ground for cellular engineering beyond the phenotypes discussed here. While genetic modification of plants or animals requires the maintenance of whole-organism viability, cell line development requires that we maintain only a subset of desirable cellular pathways and phenotypes. This system allows for more drastic remodeling of cellular metabolism and other pathways, which would normally be prohibitive to whole-organism growth, and provides intriguing options for genome minimization. While other aspects of the technology, including medium development, tissue engineering, and bioreactor design, must also progress, the demand for a vast array of improved cellular traits, together with the power and flexibility of modern cell engineering tools, creates a compelling use case in cultivated meat research and development.

## Acknowledgments

Funding was provided through the Cellulaire Agricultuur Netherlands National Growth Fund program (https://en.cellulaireagricultuur.nl/) and the 10.13039/100000199US Department of Agriculture (2021-69012-35978).

## Declaration of interests

A.J.S. is CSO of Deco Labs, Inc., a company developing enabling technologies for the cellular agriculture industry. D.L.K. is an advisor for Deco Labs, Inc. A.J.S. and D.L.K. are inventors on patent applications which involve genetic engineering for cultivated meat (US17/615,578 and US18/252,714).
